# Association of Cigarette Smoking and Alcohol Consumption With Subsequent Mortality Among Black Breast Cancer Survivors in New Jersey

**DOI:** 10.1001/jamanetworkopen.2022.52371

**Published:** 2023-01-24

**Authors:** Nur Zeinomar, Bo Qin, Saber Amin, Yong Lin, Baichen Xu, Dhanya Chanumolu, Coral O. Omene, Karen S. Pawlish, Kitaw Demissie, Christine B. Ambrosone, Chi-Chen Hong, Elisa V. Bandera

**Affiliations:** 1 Cancer Epidemiology and Health Outcomes, Rutgers Cancer Institute of New Jersey, New Brunswick; 2 Division of Medical Oncology, Department of Medicine, Rutgers Robert Wood Johnson Medical School, New Brunswick, New Jersey; 3 Department of Biostatistics and Epidemiology, Rutgers School of Public Health, Piscataway, New Jersey; 4 Cancer Epidemiology Services, New Jersey State Cancer Registry, New Jersey Department of Health, Trenton; 5 Department of Epidemiology and Biostatistics, SUNY Downstate School of Public Health, Brooklyn, New York; 6 Department of Cancer Prevention and Control, Roswell Park Comprehensive Cancer Center, Buffalo, New York

## Abstract

**Importance:**

There are limited data about how lifestyle factors are associated with breast cancer prognosis among Black or African American women because most of the evidence is based on studies of White breast cancer survivors.

**Objective:**

To examine the association of prediagnostic cigarette smoking and alcohol consumption with all-cause mortality and breast cancer–specific mortality in a cohort of Black breast cancer survivors.

**Design, Setting, and Participants:**

This population-based cohort study included 1926 Black or African American breast cancer survivors who received a diagnosis from June 6, 2005, to May 21, 2019, identified in 10 counties in New Jersey through rapid case ascertainment by the New Jersey State Cancer Registry. Statistical analysis was conducted from January 1, 2021, to August 1, 2022.

**Exposures:**

Information on prediagnostic cigarette smoking, alcohol consumption, and additional covariates was collected during in-person interviews. The covariates examined included smoking status at the time of breast cancer diagnosis (currently smoking at the time of breast cancer diagnosis, formerly smoking, or never smoking), smoking duration (number of years smoking), smoking intensity (cigarettes smoked per day), number of pack-years of smoking, and regular alcohol consumption the year before diagnosis (categorized as nondrinkers, ≤3 drinks per week, or >3 drinks per week).

**Main Outcomes and Measures:**

Primary outcomes included breast cancer–specific mortality and all-cause mortality.

**Results:**

Among the 1926 women in the study, the mean (SD) age at breast cancer diagnosis was 54.4 (10.8) years. During 13 464 person-years of follow-up (median follow-up, 6.7 years [range, 0.5-16.0 years]), there were 337 deaths, of which 187 (55.5%) were breast cancer related. Compared with never smokers, current smokers at the time of breast cancer diagnosis had a 52% increased risk for all-cause mortality (hazard ratio [HR], 1.52; 95% CI, 1.15-2.02), which was most pronounced for those with 10 or more pack-years of smoking (HR, 1.84; 95% CI, 1.34-2.53). Similar findings were observed for breast cancer–specific mortality (current smokers vs never smokers: HR, 1.27; 95% CI, 0.87-1.85), although they were not statistically significant. There was no statistically significant association between alcohol consumption and all-cause mortality (>3 drinks per week vs nondrinkers: HR, 1.05; 95% CI, 0.73-1.51) or breast cancer–specific mortality (>3 drinks per week vs nondrinkers: HR, 1.06; 95% CI, 0.67-1.67).

**Conclusions and Relevance:**

This population-based cohort study of Black breast cancer survivors suggests that current smoking at the time of diagnosis was associated with an increased risk of all-cause mortality, particularly among women with greater pack-years of smoking.

## Key Points

**Question:**

What is the association of prediagnostic cigarette smoking and alcohol consumption with all-cause and breast cancer–specific mortality among Black or African American breast cancer survivors?

**Findings:**

In this population-based cohort study of 1926 Black breast cancer survivors, current smoking at the time of breast cancer diagnosis was associated with a 52% increased risk for all-cause mortality compared with never smoking. This association was most pronounced for women with greater pack-years of smoking and who regularly consumed alcohol.

**Meaning:**

This study suggests that smoking at the time of diagnosis was associated with a higher risk of mortality among Black breast cancer survivors.

## Teaser

This cohort study uses data from the Women’s Circle of Health Follow-up Study to examine the association of prediagnostic cigarette smoking and alcohol consumption with all-cause mortality and breast cancer–specific mortality in a cohort of Black breast cancer survivors.

## Introduction

Despite advances in treatment and decreasing cancer mortality rates, survival disparities for breast cancer persist.^1,2,3^ Having a healthy lifestyle that includes not smoking and limited alcohol consumption has been associated with improved overall survival for women with breast cancer.^4^ Although smoking at the time of a breast cancer diagnosis is consistently associated with increased risk of breast cancer–specific and all-cause mortality,^5,6,7^ it is unclear whether associations vary by race and ethnicity. Only 3 studies have examined this association for Black or African American breast cancer survivors.^8,9,10^ The Carolina Breast Cancer Study^9^ and a pooled study of over 10 000 breast cancer survivors in California^10^ reported an elevated risk of breast cancer–specific mortality for African American women who were current or ever smokers (vs never smokers), but not for non–African American women.^10^ Conversely, another study reported smoking to be associated with worse survival among White breast cancer survivors, while Black women had similar survival regardless of smoking status.^8^

In contrast to smoking, less is known about how alcohol use is associated with breast cancer prognosis,^11^ despite alcohol use being an established risk factor for breast cancer incidence.^11,12,13,14^ The evidence linking the association between alcohol intake and breast cancer prognosis has been inconclusive, likely owing to different prognostic outcomes evaluated, variation in drinking patterns, differences in exposure assessment, and failure to adjust for important confounders.^15,16,17,18^ Moreover, most studies have been conducted with White women, so there is limited information on how drinking may be associated with breast cancer prognosis for Black women. Only 1 study presented stratified results by race and ethnicity, and it was limited to models examining lifetime wine consumption and breast cancer–specific mortality, for which no evidence of increased risk was observed.^19^

Furthermore, while smoking prevalence has decreased among cancer survivors,^20^ there has been a persistent trend of increased alcohol consumption among cancer survivors over the past decade.^20,21^ In addition, there are known differences across racial and ethnic groups in patterns of behaviors and adherence to healthy lifestyle recommendations.^22^ Understanding how these modifiable lifestyle factors are associated with breast cancer prognosis for Black or African American women (referred to hereafter as *Black women*) is important for clinical recommendations and management after a breast cancer diagnosis, especially given the 41% higher mortality rate for Black women compared with their White counterparts after a breast cancer diagnosis.^1^ To this end, we examined the association of cigarette smoking and alcohol consumption with all-cause and breast cancer–specific mortality among participants in the Women’s Circle of Health Follow-up Study (WCHFS), an ongoing population-based cohort study of Black breast cancer survivors in New Jersey.

## Methods

### Study Population

The WCHFS^23^ includes 1928 Black breast cancer survivors who received a diagnosis from June 6, 2005, to May 21, 2019, identified in 10 counties in New Jersey through rapid case ascertainment by the New Jersey State Cancer Registry (NJSCR), as previously described.^23,24^ In brief, eligibility criteria include women who self-identify as Black or African American; have incident, histologically confirmed ductal carcinoma in situ or invasive breast cancer; are aged 20 to 75 years at breast cancer diagnosis; are English speaking; and have no prior history of cancer except nonmelanoma skin cancer. As previously described, distributions of tumor stage and grade were similar for participants and all eligible breast cancer cases identified by the NJSCR, suggesting that the tumor characteristics of WCHFS participants are representative of Black breast cancer survivors in New Jersey.^23^ All participants provided written informed consent. The study protocol was approved by the institutional review boards at Rutgers University and Roswell Park Comprehensive Cancer Center. This study followed the Strengthening the Reporting of Observational Studies in Epidemiology (STROBE) reporting guideline for cohort studies.

At the initial home visit, approximately 10 months after breast cancer diagnosis, participants completed an interviewer-administered questionnaire that collected information on sociodemographic factors and breast cancer risk factors, including family history of breast cancer, reproductive factors, hormone use, medical history, medication use, and dietary and lifestyle factors (alcohol intake, smoking history, and physical activity). Anthropometric measurements (weight, height, and body composition), blood pressure measurements, and biospecimens were also collected during the home visits at the time of interview. Information on clinical and tumor characteristics, such as stage and hormone receptor status (eg, estrogen receptor [ER]), were abstracted from pathology reports and NJSCR files. In the present analyses, we excluded 2 women who had missing information on alcohol consumption or smoking status, leaving 1926 women to be included in this study.

### Exposure Assessment

For cigarette smoking, we defined smokers as those who reported smoking at least 1 cigarette per day for 1 year and current smokers as women who reported smoking at the time of their breast cancer diagnosis (hereafter referred to as *current smokers*). Smoking duration was calculated from the age the participants started smoking to the age the participant quit smoking, accounting for periods of time that the women did not smoke. Smoking intensity was caclulated as the mean number of cigarettes smoked per day, during the period when women smoked. We estimated pack-years as the product of smoking intensity (cigarette packs smoked per day) by smoking duration (years smoked). For former and current smokers, we categorized pack-years (<10 or ≥10 pack-years of smoking), smoking duration (<25 or ≥25 years), and smoking intensity (<8 or ≥8 cigarettes per day). For former smokers, we also examined the recency of smoking cessation (≤10 or >10 years).

We asked Black women about their regular consumption of alcoholic beverages during the year before diagnosis. For drinkers, we collected additional information about the amount, type (beer, wine, or liquor), and frequency of alcohol consumed. We defined nondrinkers as women who did not report drinking or drank zero alcoholic beverages per week. For regular drinkers, we estimated the number of alcoholic drinks consumed per week as 3 or less or more than 3 drinks per week, where 1 serving was defined as 355 mL (12 fl oz) of beer, 177 mL (6 fl oz) of wine (1 medium glass), or 45 mL (1.5 fl oz) of liquor (1 shot). We estimated alcoholic drinks per week as the sum of the intake of each of the 3 different types of alcoholic beverages consumed. Because the overall drinking level was low in our cohort, we could not examine drinking levels above the current recommendations of 1 drink per day for women.^25^

### Outcome Ascertainment

Primary outcomes of interest included breast cancer–specific and all-cause mortality. Outcomes were ascertained through linkage with the NJSCR, which collected information on vital status, date of death, and cause of death. The NJSCR is updated using multiple sources, including the National Death Index, hospital discharge files, Medicare and Medicaid files, and Social Security administrative data, allowing for virtually complete ascertainment of mortality.

### Statistical Analysis

Statistical analysis was conducted from January 1, 2021, to August 1, 2022. We used Cox proportional hazards regression models to estimate hazard ratios (HRs) and corresponding 95% CIs for the association of each smoking and alcohol variable with all-cause mortality. We estimated the number of person-years from date of diagnosis to death or the end of follow-up (September 3, 2021). When examining breast cancer–specific mortality, we used the Fine-Gray subdistribution hazard model^26^ to account for competing risks, and death from other causes was considered the competing event. We assessed the proportional hazards assumption through Schoenfeld residuals. We selected covariates for the final model based on directed acyclic graphs and the prior literature. These covariates included age at diagnosis, tumor stage, body mass index (BMI; calculated as weight in kilograms divided by height in meters squared), educational level, inflation-adjusted household income, marital status, menopausal status, physical activity, alcohol consumption (for smoking variables), and smoking status (for alcohol consumption). For models examining recency of smoking cessation, the covariates included age at diagnosis, tumor stage, waist-to-hip ratio, history of diabetes, history of hypertension, educational level, inflation-adjusted household income, and physical activity. Treatment variables (type of surgery, chemotherapy, radiotherapy, and hormone therapy) were considered in the directed acyclic graphs; they were not included in the final models because they were not determined to be confounders. As a sensitivity analysis, we adjusted for treatment variables that were associated with our exposures (surgery, chemotherapy, and radiotherapy); this adjustment did not substantially change our results. We also examined alcohol and smoking combined by examining the statistical interaction of the 2 variables on the multiplicative and additive scale. We assessed a multiplicative interaction by including a cross-product term in the model and assessing the corresponding β coefficient using the Wald test, and we assesed an additive interaction by estimating the relative excess risk due to the interaction.^27^ For all variables, we examined a multiplicative interaction by age group (<40, 40 to ≤60, and >60 years), menopausal status (premenopausal or postmenopausal), ER status, BMI (<30 and ≥30), waist-to-hip ratio (≤0.85 and >0.85), and family history of breast cancer (yes or no), as well as stratified models. We performed sensitivity analyses by excluding ductal carcinoma in situ cases and excluding women with a diagnosis of stage IV tumors. We also performed sensitivity analyses estimating follow-up time starting from date of interview (instead of date of diagnosis) to account for potential immortal time bias; this did not change our estimates. All statistical tests were 2-sided, and *P* < .05 was considered statistically significant. All statistical analyses were performed in SAS, version 9.4 (SAS Institute Inc).

## Results

Among the 1926 women in the study, the mean (SD) age at breast cancer diagnosis was 54.4 (10.8) years (Table 1). During 13 464 person-years of follow-up (median follow-up, 6.7 years [range, 0.5-16.0 years]), we observed 337 deaths, of which 187 (55.5%) were breast cancer specific. Other causes of death included other cancers, not including breast cancer (39 of 337 [11.6%]); cardiovascular, vascular, or atherosclerotic disease (32 of 337 [9.5%]); infections including COVID-19 (12 of 337 [3.6%]); respiratory conditions (10 of 337 [3.0%]); kidney failure (5 of 337 [1.5%]); metabolic disorder, diabetes, or obesity-related death (5 of 337 [1.5%]); pregnancy-related death (5 of 337 [1.5%]); and other causes (42 of 337 [12.5%]). Descriptive characteristics of the cohort overall and by smoking status are shown in Table 1. Of the 1926 women in this study, 16.5% (n = 317) reported smoking at the time of their breast cancer diagnosis, 24.3% (n = 468) reported former smoking, and more than half reported never smoking (59.2% [n = 1141]). Less than half of the women (41.5% [n = 799]) reported regularly consuming alcohol in the year before their breast cancer diagnosis. Of regular drinkers, 70.6% (n = 564) reported consuming 3 or fewer alcoholic drinks per week, and less than one-third of regular drinkers drank more than 3 drinks per week (28.3% [n = 226]). Current smokers consumed more alcoholic beverages per week (19.9% [63 of 317] consuming >3 drinks per week) than did former smokers (14.3% [67 of 468] consuming >3 drinks per week) or never smokers (8.4% [96 of 1141] consuming >3 drinks per week). Estrogen receptor status was similar across levels of smoking status, with 70.3% of the overall cohort (n = 1354) having an ER-positive tumor.

**Table 1.** Descriptive Characteristics of Participants in the Women’s Circle of Health Follow-up Study, by Cigarette Smoking

Characteristic	Participants, No. (%)				*P* value^a^
	Never smokers (n = 1141)	Former smokers (n = 468)	Current smokers (n = 317)	Total (N = 1926)	
Age at breast cancer diagnosis, mean (SD), y	53.0 (11.2)	58.3 (9.4)	53.9 (9.6)	54.4 (10.8)	.001
Age at breast cancer diagnosis, y					
<40	149 (13.1)	18 (3.8)	26 (8.2)	193 (10.0)	.001
40 to ≤60	669 (58.6)	243 (51.9)	214 (67.5)	1126 (58.5)	
>60	323 (28.3)	207 (44.2)	77 (24.3)	607 (31.5)	
Menopausal status					
Premenopausal	526 (46.1)	112 (23.9)	109 (34.4)	747 (38.8)	.001
Postmenopausal	615 (53.9)	356 (76.1)	208 (65.6)	1179 (61.2)	
Smoking					
Never smokers	1141 (100)	NA	NA	1141 (59.2)	NA
Former smokers, pack-years					
<10	NA	284 (60.7)	NA	284 (14.7)	
≥10	NA	184 (39.3)	NA	184 (9.6)	
Current smokers, pack-years					
<10	NA	NA	128 (40.4)	128 (6.6)	
≥10	NA	NA	189 (59.6)	189 (9.8)	
Smoking intensity					
Never smokers	1141 (100)	NA	NA	1141 (59.2)	NA
Former smokers, cigarettes/d					
<8	NA	232 (49.6)	NA	232 (12.0)	
≥8	NA	236 (50.4)	NA	236 (12.3)	
Current smokers, cigarettes/d					
<8	NA	NA	144 (45.4)	144 (7.5)	
≥8	NA	NA	173 (54.6)	173 (9.0)	
Smoking duration					NA
Never smokers	1141 (100)	NA	NA	1141 (59.2)	
Former smokers, y					
<25	NA	316 (67.5)	NA	316 (16.4)	
≥25	NA	152 (32.5)	NA	152 (7.9)	
Current smokers, y					
<25	NA	NA	69 (21.8)	69 (3.6)	
≥25	NA	NA	248 (78.2)	248 (12.9)	
Recency of cessation for former smokers, y					
≤10	NA	152 (32.5)	NA	152 (7.9)	NA
>10	NA	316 (67.5)	NA	316 (16.4)	
Age at smoking initiation, mean (SD), y	NA	19.5 (5.7)	19.2 (5.8)	19.4 (5.8)	.47
Smoking duration, mean (SD), y	NA	19.5 (12.0)	33.3 (11.2)	25.1 (13.5)	<.001
Smoking intensity, mean (SD), cigarettes/d	NA	10.3 (8.5)	9.4 (6.1)	10.0 (7.7)	.09
Alcohol consumption					
Nondrinker^b^	716 (62.8)	263 (56.2)	148 (46.7)	1127 (58.5)	.001
≤3 drinks/wk	324 (28.4)	137 (29.3)	103 (32.5)	564 (29.3)	
>3 drinks/wk	96 (8.4)	67 (14.3)	63 (19.9)	226 (11.7)	
Missing	5 (0.4)	1 (0.2)	3 (0.9)	9 (0.5)	
BMI					
<30	511 (44.8)	173 (37.0)	160 (50.5)	844 (43.8)	.001
≥30	628 (55.0)	294 (62.8)	156 (49.2)	1078 (56.0)	
Missing	2 (0.2)	1 (0.2)	1 (0.3)	4 (0.2)	
BMI, mean (SD)	31.7 (6.9)	33.1 (7.0)	30.9 (7.3)	31.9 (7.0)	.001
Waist-to-hip ratio					
≤0.85	383 (33.6)	110 (23.5)	83 (26.2)	576 (29.9)	.001
>0.85	729 (63.9)	348 (74.4)	224 (70.7)	1301 (67.5)	
Missing	29 (2.5)	10 (2.1)	10 (3.2)	49 (2.5)	
Physical activity, MET h/wk					
<11.7	350 (30.7)	154 (32.9)	131 (41.3)	635 (33.0)	.007
11.8-37.9	402 (35.2)	148 (31.6)	91 (28.7)	641 (33.3)	
≥38	389 (34.1)	163 (34.8)	94 (29.7)	646 (33.5)	
Missing	0	3 (0.6)	1 (0.3)	4 (0.2)	
Physical activity, mean (SD), MET h/wk	38.5 (49.7)	39.1 (46.9)	34.2 (48.2)	37.9 (48.8)	.32
Educational level					
High school graduate or less	391 (34.3)	181 (38.7)	164 (51.7)	736 (38.2)	.001
Some college or vocational school	346 (30.3)	155 (33.1)	104 (32.8)	605 (31.4)	
Bachelor’s or graduate degree	403 (35.3)	132 (28.2)	48 (15.1)	583 (30.3)	
Missing	1 (0.1)	0	1 (0.3)	2 (0.1)	
Household income per capita, adjusted for inflation, $					
<15 000	349 (30.6)	137 (29.3)	144 (45.4)	630 (32.7)	.001
15 000-29 999	376 (33.0)	132 (28.2)	97 (30.6)	605 (31.4)	
≥30 000	331 (29.0)	162 (34.6)	56 (17.7)	549 (28.5)	
Unknown	85 (7.4)	37 (7.9)	20 (6.3)	142 (7.4)	
Insurance					
Private	691 (60.6)	255 (54.5)	130 (41.0)	1076 (55.9)	.001
Medicare or Medicaid	254 (22.3)	153 (32.7)	134 (42.3)	541 (28.1)	
Uninsured	115 (10.1)	39 (8.3)	43 (13.6)	197 (10.2)	
Other or missing	81 (7.1)	21 (4.5)	10 (3.2)	112 (5.8)	
Marital status					
Married or living as married	461 (40.4)	169 (36.1)	79 (24.9)	709 (36.8)	.001
Widow	101 (8.9)	69 (14.7)	19 (6.0)	189 (9.8)	
Divorced or separated	263 (23.0)	121 (25.9)	84 (26.5)	468 (24.3)	
Single or never married	314 (27.5)	107 (22.9)	135 (42.6)	556 (28.9)	
Missing	2 (0.2)	2 (0.4)	0	4 (0.2)	
Estrogen receptor status					
Positive	806 (70.6)	324 (69.2)	224 (70.7)	1354 (70.3)	.63
Negative	294 (25.8)	132 (28.2)	89 (28.1)	515 (26.7)	
Missing	41 (3.6)	12 (2.6)	4 (1.3)	57 (3.0)	
Tumor stage					
0	195 (17.1)	107 (22.9)	42 (13.2)	344 (17.9)	.001
I	408 (35.8)	183 (39.1)	104 (32.8)	695 (36.1)	
II	368 (32.3)	124 (26.5)	115 (36.3)	607 (31.5)	
III and IV	147 (12.9)	46 (9.8)	51 (16.1)	244 (12.7)	
Missing	23 (2.0)	8 (1.7)	5 (1.6)	36 (1.9)	
Breast cancer subtype					
Luminal A	538 (47.2)	220 (47.0)	166 (52.4)	924 (48.0)	.38
ERBB2 (formerly HER2) positive	208 (18.2)	65 (13.9)	54 (17.0)	327 (17.0)	
Triple negative	191 (16.7)	85 (18.2)	57 (18.0)	333 (17.3)	
Unknown or missing	204 (17.9)	98 (20.9)	40 (12.6)	342 (17.8)	
Surgery					
No surgery	30 (2.6)	15 (3.2)	21 (6.6)	66 (3.4)	.001
Lumpectomy	576 (50.5)	267 (57.1)	136 (42.9)	979 (50.8)	
Mastectomy	535 (46.9)	185 (39.5)	160 (50.5)	880 (45.7)	
Unknown or missing	0	1 (0.2)	0	1 (0.1)	
Chemotherapy					
No	495 (43.4)	247 (52.8)	132 (41.6)	874 (45.4)	.001
Yes	644 (56.4)	219 (46.8)	184 (58.0)	1047 (54.4)	
Unknown or missing	2 (0.2)	2 (0.4)	1 (0.3)	5 (0.3)	
Radiotherapy					
No	376 (33.0)	134 (28.6)	117 (36.9)	627 (32.6)	.04
Yes	765 (67.0)	333 (71.2)	196 (61.8)	1294 (67.2)	
Unknown or missing	0	1 (0.2)	4 (1.3)	5 (0.3)	
Hormone therapy					
No	399 (35.0)	169 (36.1)	121 (38.2)	689 (35.8)	.48
Yes	741 (64.9)	296 (63.2)	192 (60.6)	1229 (63.8)	
Unknown or missing	1 (0.09)	3 (0.6)	4 (1.3)	8 (0.4)	
History of diabetes					
No	805 (70.6)	277 (59.2)	209 (65.9)	1291 (67.0)	.001
Yes	222 (19.5)	136 (29.1)	72 (22.7)	430 (22.3)	
Unknown or missing	114 (10.0)	55 (11.8)	36 (11.4)	205 (10.6)	
History of hypertension					
No	370 (32.4)	107 (22.9)	75 (23.7)	552 (28.7)	.001
Yes	657 (57.6)	306 (65.4)	206 (65.0)	1169 (60.7)	
Unknown or missing	114 (10.0)	55 (11.8)	36 (11.4)	205 (10.6)	
First-degree family history of breast cancer					
No	916 (80.3)	374 (79.9)	255 (80.4)	1545 (80.2)	.85
Yes	198 (17.4)	87 (18.6)	54 (17.0)	339 (17.6)	
Missing	27 (2.4)	7 (1.5)	8 (2.5)	42 (2.2)	

Abbreviations: BMI, body mass index (calculated as weight in kilograms divided by height in meters squared); MET, metabolic equivalent task; NA, not applicable.

^a^ From the χ^2^ test, *t* test, or analysis of variance when appropriate.

^b^ Nondrinkers defined as women who did not report drinking or drank zero alcoholic beverages per week.

Smoking at the time of diagnosis was associated with an increased risk of all-cause mortality; compared with never smokers, current smokers had a 52% increased risk for all-cause mortality (HR, 1.52; 95% CI, 1.15-2.02) after adjusting for covariates (Table 2). When we examined this association within levels of pack-years, we found an 84% increased risk of all-cause mortality for current smokers who smoked 10 or more cumulative pack-years (HR, 1.84; 95% CI, 1.34-2.53) and no association for current smokers who smoked fewer than 10 pack-years (HR, 1.06; 95% CI, 0.67-1.66). We also observed an increased risk of breast cancer–specific mortality for current smokers, although less substantial than all-cause mortality and with wider 95% CIs, likely due to limited power. Compared with never smokers, current smokers had a 27% increased risk for breast cancer–specific mortality (HR, 1.27; 95% CI, 0.87-1.85), which was greater for women who were heavier smokers, at 41% (≥10 pack-years: HR, 1.41; 95% CI, 0.88-2.26). In addition, recency of smoking cessation was not associated with either all-cause or breast cancer–specific mortality. The overall findings for smoking variables were similar in sensitivity analyses of models excluding stage IV tumors (eTable 1 in Supplement 1), models limited to only invasive breast cancers (eTable 2 in Supplement 1), and models adjusted for treatment factors including chemotherapy, surgery, and radiotherapy (current smokers [vs never smokers]: HR, 1.45; 95% CI, 1.09-1.93).

**Table 2.** Associations of Cigarette Smoking With Mortality After First Breast Cancer in the Women’s Circle of Health Follow-up Study

Characteristic	All-cause mortality			Breast cancer–specific mortality		
	Person-years	Total deaths	HR (95% CI)	Person-years	Breast cancer deaths	HR (95% CI)
Smoking status^a^						
Never smokers	7897.2	168	1 [Reference]	7897.2	102	1 [Reference]
Former smokers	3261.5	82	1.25 (0.95-1.65)	3261.5	41	1.22 (0.82-1.81)
Current smokers	2094.2	80	1.52 (1.15-2.02)	2094.2	42	1.27 (0.87-1.85)
No. of pack-years of smoking^a^						
Never smokers	7897.2	168	1 [Reference]	7897.2	102	1 [Reference]
Former smokers						
<10	1951.2	49	1.19 (0.86-1.65)	1951.2	27	1.21 (0.77-1.89)
≥10	1310.3	33	1.39 (0.94-2.05)	1310.3	14	1.24 (0.67-2.31)
Current smokers						
<10	886.4	23	1.06 (0.67-1.66)	886.4	15	1.06 (0.62-1.81)
≥10	1207.8	57	1.84 (1.34-2.53)	1207.8	27	1.41 (0.88-2.26)
Duration of smoking, y^a^						
Never smokers	7897.2	168	1 [Reference]	7897.2	102	1 [Reference]
Former smokers						
<25	2244.4	48	1.08 (0.78-1.50)	2244.4	24	1.01 (0.63-1.64)
≥25	1017.1	34	1.67 (1.13-2.47)	1017.1	17	1.87 (1.06-3.29)
Current smokers						
<25	448.2	17	1.43 (0.85-2.42)	448.2	8	0.80 (0.39-1.65)
≥25	1645.9	63	1.56 (1.15-2.11)	1645.9	34	1.48 (0.97-2.25)
Intensity of smoking^a^						
Never smokers	7897.2	168	1 [Reference]	7897.2	102	1 [Reference]
Former smokers, cigarettes/d						
<8	1583.4	41	1.22 (0.86-1.74)	1583.4	22	1.26 (0.78-2.03)
≥8	1678.1	41	1.28 (0.90-1.82)	1678.1	19	1.17 (0.68-2.03)
Current smokers, cigarettes/d						
<8	982.1	31	1.35 (0.91-2.02)	982.1	19	1.34 (0.82-2.21)
≥8	1112.1	49	1.65 (1.18-2.30)	1112.1	23	1.21 (0.74-2.00)
Recency of cessation^b^						
Never smokers	6889.7	136	1 [Reference]	6889.7	81	1 [Reference]
Current smokers	1830.3	65	1.55 (1.15-2.10)	1830.3	32	1.21 (0.79-1.84)
Former smokers, y						
≤10	856.3	23	1.40 (0.89-2.20)	856.3	11	1.26 (0.63-2.51)
>10	1878.6	44	1.19 (0.84-1.70)	1878.6	23	1.22 (0.74-2.00)

Abbreviation: HR, hazard ratio.

^a^ Cox proportional hazards regression models adjusted for age at diagnosis, tumor stage, body mass index, alcohol consumption, educational level, household income, marital status, menopausal status, and physical activity. Competing risk models (Fine-Gray subdistribution hazard model) were used for breast cancer–specific mortality.

^b^ Cox proportional hazards regression models adjusted for age at diagnosis, tumor stage, waist-to-hip ratio, history of diabetes, history of hypertension, educational level, household income, and physical activity. Competing risk models (Fine-Gray subdistribution hazard model) were used for breast cancer–specific mortality.

**Figure 1.**
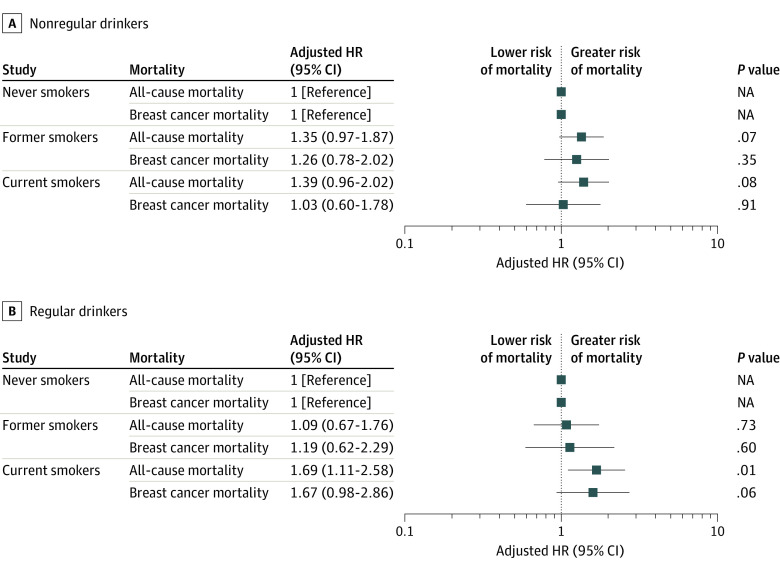
Association of Smoking Status With All-Cause Mortality and Breast Cancer–Specific Mortality, by Drinking Status Hazard ratios (HRs) and 95% CIs were estimated using Cox proportional hazards regression models adjusted for age at diagnosis, tumor stage, body mass index, educational level, household income, marital status, menopausal status, and physical activity. NA indicates not applicable.

As shown in Table 3, regular consumption of alcohol the year before diagnosis was not associated with all-cause mortality (>3 drinks per week vs nondrinkers: HR, 1.05; 95% CI, 0.73-1.51) or breast cancer–specific mortality mortality (>3 drinks per week vs nondrinkers: HR, 1.06; 95% CI, 0.67-1.67). To further assess the combination of smoking and drinking combined, we examined the interaction of the 2 exposures on a multiplicative and additive scale. Although the multiplicative and additive interaction of smoking and alcohol were not statistically significant, compared with never smokers who regularly drank alcohol, current smokers who regularly drank alcohol had a 69% increased risk of all-cause mortality (HR, 1.69; 95% CI, 1.11-2.58), whereas the association for current smokers who did not regularly drink alcohol (compared with never smokers who did not regularly drink alcohol) was less substantial (HR, 1.39; 95% CI, 0.96-2.02) (Figure 1). We observed similar associations for breast cancer–specific mortality, with a 67% increased risk for current smokers who regularly drank alcohol compared with never smokers who regularly drank alcohol (HR, 1.67; 95% CI, 0.98-2.86), but no association for those who were not regular drinkers (Figure 1).

**Table 3.** Associations of Prediagnostic Alcohol Consumption With Mortality After First Breast Cancer in the Women's Circle of Health Follow-up Study

Total alcoholic drinks per week	All-cause mortality			Breast cancer–specific mortality		
	Person-years	Total deaths	HR (95% CI)	Person-years	Breast cancer deaths	HR (95% CI)^a^
						
Nondrinker^b^	7928.6	211	1 [Reference]	7928.6	114	1 [Reference]
≤3	3870.7	81	0.83 (0.64-1.09)	3870.7	47	0.79 (0.55-1.12)
>3	1453.6	38	1.05 (0.73-1.51)	1453.6	24	1.06 (0.67-1.67)

Abbreviation: HR, hazard ratio.

^a^ Cox proportional hazards regression models adjusted for age at diagnosis, tumor stage, body mass index, cigarette smoking, educational level, household income, marital status, menopausal status, and physical activity. Competing risk models (Fine-Gray subdistribution hazard model) were used for breast cancer–specific mortality.

^b^ Defined as women who did not report drinking or drank zero alcoholic beverages per week.

**Figure 2.**
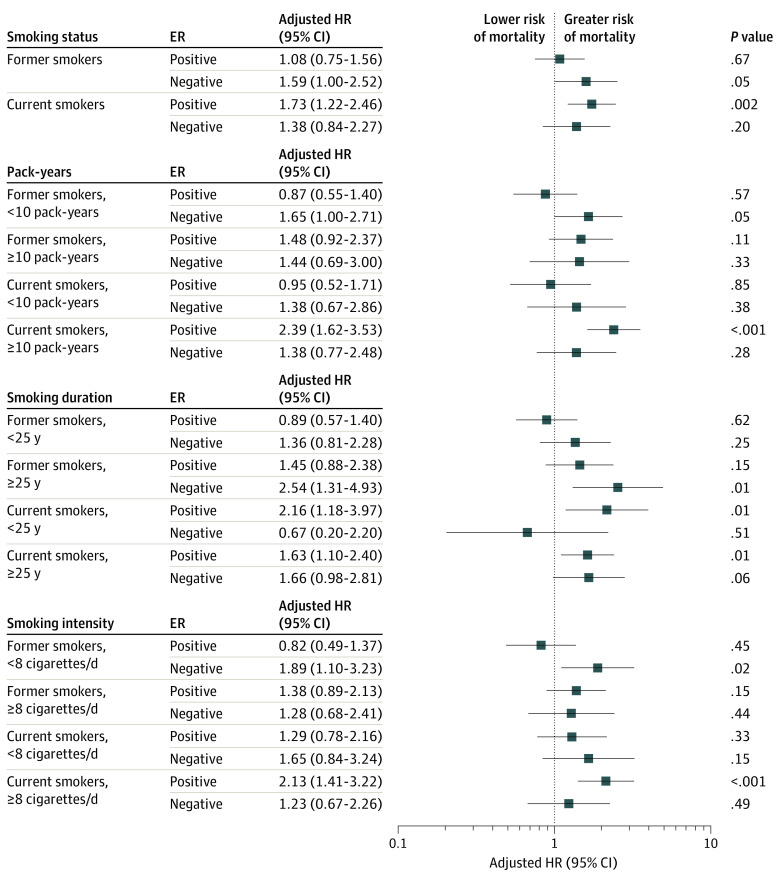
Association of Smoking Variables With All-Cause Mortality, Stratified by Estrogen Receptor (ER) Status The reference category is never smokers. Hazard ratios (HRs) and 95% CIs were estimated using Cox proportional hazards regression models adjusted for age at diagnosis, tumor stage, body mass index, alcohol consumption, educational level, household income, marital status, menopausal status, and physical activity.

We did not find evidence of a multiplicative interaction with any of the smoking or alcohol variables and ER status, age group, menopausal status, BMI, waist-to-hip ratio, or family history of breast cancer. In stratified analyses by ER status, we observed an elevated risk of all-cause mortality for both ER-positive and ER-negative tumors for current smokers compared with never smokers (ER positive: HR, 1.73; 95% CI, 1.22-2.46; ER negative: HR, 1.38; 95% CI, 0.84-2.27), although this finding was not statistically significant for ER-negative tumors (Figure 2).

## Discussion

In this large population-based cohort study of Black breast cancer survivors, smoking at the time of diagnosis was associated with an increased risk of all-cause mortality. Our findings of elevated risk of all-cause mortality for current smokers are consistent with prior findings from studies primarily comprising White women.^6,28^ Three prior studies examined these associations for Black women^8,9,10^ but had limited information on detailed smoking behaviors and did not examine smoking and alcohol consumption combined. John et al^10^ reported a 47% increased risk of all-cause mortality for African American women with ER-positive or progesterone receptor–positive tumors who ever smoked (compared with never smokers) but no increased risk for non-Hispanic White women or African American ever smokers with ER-negative tumors. On the other hand, 2 studies reported an association of elevated risk of all-cause mortality among current smokers, but only for non–African American women.^8,9^

Parada et al^9^ reported an association of current smoking and all-cause mortality for current smokers, but only for women who survived at least 5 years after diagnosis; however, when stratified by race, this association was not observed among African American women. This study reported a 69% increased risk of breast cancer–specific mortality for African American women who survived at least 5 years after diagnosis, but not for non–African American women. This finding contrasts with our findings of an elevated risk of all-cause mortality but not breast cancer–specific mortality for current smokers vs never smokers. The study by Parada et al^9^ had a longer follow-up time than our study (median follow-up, 13.6 vs 6.7 years), so our findings for breast cancer–specific mortality may be limited by the fewer number of deaths and shorter follow-up.

In our study, we found a 41% increased risk for breast cancer–specific mortality for current smokers with 10 or more pack-years (vs never smokers), but the 95% CI included the null value. In a study using large administrative databases in Florida, current smoking was associated with worse survival for White and Hispanic women but not for Black women^8^; however, that study had information only on smoking status and intensity, had a short mean follow-up of 4.9 years, and did not directly ask women about their smoking history. Although an overall association was not observed between smoking and breast cancer–specific mortality, possibly due to insufficient follow-up time, the association between smoking and poorer prognosis for breast cancer survivors is biologically plausible, and several mechanisms have been proposed. Smoking can enhance the metastatic ability of breast cancer cells and induce tumor growth,^29,30^ is associated with other comorbidities that affect survival, and may increase resistance to cancer therapies.^31^

Our finding of no association between alcohol consumption and all-cause mortality or breast cancer–specific mortality is consistent with prior literature.^18,19,32,33^ However, our cohort had low levels of alcohol consumption. A meta-analysis reported an association between 20 g per day of alcohol consumption, but not lower levels of consumption, and increased breast cancer mortality.^15^ In addition, there are known differences in alcohol consumption patterns between racial and ethnic subgroups,^34,35^ with lower levels of drinking reported for Black women compared with White women.

Even though we did not observe an association with drinking and all-cause mortality or breast cancer–specific mortality, we did find a stronger association with mortality for current smokers who regularly drank alcohol. In stratified analyses, we observed a 69% increased risk for all-cause mortality and a 67% increased risk for breast cancer–specific mortality for women who both regularly drank and were current smokers compared with never smokers who regularly drank, but we observed no association for current smokers who were nonregular drinkers. To our knowledge, no other study has examined the association of mortality with smoking and drinking combined among Black women. Consistent with our findings, Din et al^36^ reported a 92% increased risk of breast cancer–specific mortality among current smokers who consumed alcohol, but the study did not report stratified models. In addition, similar to the differences in alcohol patterns by race and ethnicity, a recent study using National Health and Nutrition Examination Survey data for female cancer survivors reported that Black women were less likely than non-Hispanic White women to report smoking.^22^ However, Black cancer survivors with at least 1 comorbidity were more likely to smoke within their lifetime compared with non-Hispanic White survivors. Considering the high prevalence of comorbidities among Black breast cancer survivors, drinking history should be captured along with smoking history during clinical management, and there may be a benefit associated with targeted prevention efforts for women who both smoke and regularly consume alcohol. Moreover, recent alcohol consumption was found to be associated with continued smoking after a cancer diagnosis among African American cancer survivors,^37^ underscoring the importance of examining these behaviors together.

### Limitations and Strengths

Our study has some limitations. We could not assess the association of smoking or alcohol consumption after diagnosis because we had limited power given that only women recruited after 2014 (when additional funding was obtained) were invited to the follow-up study.^23^ However, of the subset of women with smoking and drinking information at follow-up, 86% of those who reported smoking since their breast cancer diagnosis were also current smokers at breast cancer diagnosis, suggesting that prediagnosis and postdiagnosis smoking are associated. We observed similar findings for alcohol consumption. We also did not have information on other types of smoking, including passive smoking, e-cigarette use, and hookah smoking. In addition, both alcohol and smoking exposures were based on self-report and may be underreported. Finally, we had limited power to examine interactions, especially for breast cancer–specific mortality or associations by breast cancer subtype.

Our study also has some strengths. A major strength is the cohort because the WCHFS is one of the few population-based epidemiologic studies available of Black breast cancer survivors that has extensive epidemiologic data, with data on outcomes after breast cancer diagnosis. This allowed us to examine the association of several smoking exposures and of alcohol and smoking combined, as well as adequately control for important confounders.

## Conclusions

Our cohort study suggests that smoking at the time of a breast cancer diagnosis is associated with an increased risk of all-cause mortality, particularly for women who also drank and had 10 or more pack-years of smoking. Although we observed a similar elevated risk associated with smoking for breast cancer–specific mortality, risk estimates were not statistically significant, likely due to limited power. Future studies among Black breast cancer survivors are warranted to understand the role of smoking and alcohol in prognosis, including its role in recurrence and mortality due to breast cancer and by tumor subtype, as well as factors associated with continued smoking. Our findings add to the evidence of the detrimental health effects of smoking and underscore the need of tailored and targeted survivorship care for breast cancer survivors, particularly women with heavier levels of smoking.

## Notes

Supplement 1. **eTable 1.** Hazard Ratios and 95% Confidence Intervals for Associations of Cigarette Smoking and Mortality After First Breast Cancer in the Women’s Circle of Health Follow-up Study (WCHFS), Excluding Stage IV Tumors **eTable 2.** Hazard Ratios and 95% Confidence Intervals for Associations of Cigarette Smoking and Mortality After First Breast Cancer in the Women’s Circle Of Health Follow-up Study (WCHFS), Excluding Ductal Carcinoma In-Situ

Supplement 2.
Data Sharing Statement
