# Supplementary material for: Association of Cigarette Smoking and Alcohol Consumption With Subsequent Mortality Among Black Breast Cancer Survivors in New Jersey
Source: JAMA Netw Open. 2023 Jan 24;6(1):e2252371. doi: 10.1001/jamanetworkopen.2022.52371 (PMC10148653; doi:10.1001/jamanetworkopen.2022.52371)
Supplement: Supplement 1. — eTable 1. Hazard Ratios and 95% Confidence Intervals for Associations of Cigarette Smoking and Mortality After First Breast Cancer in the Women’s Circle of Health Follow-up Study (WCHFS), Excluding Stage IV Tumors eTable 2. Hazard Ratios and 95% Confidence Intervals for Associations of Cigarette Smoking and Mortality After First Breast Cancer in the Women’s Circle Of Health Follow-up Study (WCHFS), Excluding Ductal Carcinoma In-Situ [file jamanetwopen-e2252371-s001.pdf]

Supplementary Online Content

Zeinomar N, Qin B, Amin S, et al. Association of cigarette smoking and alcohol consumption with subsequent mortality among Black breast cancer survivors in New Jersey. *JAMA Netw Open.* 2023;6(1):e2252371.
doi:10.1001/jamanetworkopen.2022.52371

eTable 1. Hazard Ratios and 95% Confidence Intervals for Associations of Cigarette Smoking and Mortality After First Breast Cancer in the Women's Circle of Health Follow-up Study (WCHFS), Excluding Stage IV Tumors
eTable 2. Hazard Ratios and 95% Confidence Intervals for Associations of Cigarette Smoking and Mortality After First Breast Cancer in the Women's Circle Of Health Follow-up Study (WCHFS), Excluding Ductal Carcinoma In-Situ

This supplementary material has been provided by the authors to give readers additional information about their work.

© 2023 Zeinomar N et al. *JAMA Network Open.*

eTable 1. Hazard Ratios and 95% Confidence Intervals for Associations of Cigarette Smoking and Mortality After First Breast Cancer in the Women's Circle of Health Follow-up Study (WCHFS), Excluding Stage IV Tumors

	All-cause mortality				Breast cancer–specific mortality		
	Person-years	Total deaths	HR (95% CI)		Person-years	Breast cancer deaths	HR (95% CI)
**Smoking Status** [a]							
Never smokers	7796.7	147	Reference		7796.7	85	Reference
Former smokers	3228	76	1.30 (0.97, 1.73)		3228	37	1.35 (0.88, 2.07)
Current smokers	2033.4	70	1.58 (1.17, 2.13)		2033.4	36	1.38 (0.92, 2.08)
**Pack years of smoking** [a]							
Never smokers	7796.7	147	Reference		7796.7	85	Reference
Former smokers, < 10 pack years	1924.5	43	1.20 (0.85, 1.70)		1951.2	23	1.30 (0.79, 2.14)
Former smokers, ≥ 10 pack years	1303.4	33	1.47 (0.99, 2.18)		1310.3	14	1.45 (0.78, 2.72)
Current smokers, < 10 pack years	864.6	19	0.99 (0.61, 1.62)		886.4	13	1.09 (0.61, 1.96)
Current smokers, ≥ 10 pack years	1168.8	51	2.02 (1.44, 2.82)		1207.8	23	1.62 (0.98, 2.68)
**Duration of smoking** [a]							
Never smokers	7796.7	168	Reference		7796.7	85	Reference
Former smokers, < 25 years	2222.2	43	1.09 (0.77, 1.55)		2222.2	21	1.09 (0.65, 1.84)
Former smokers, ≥ 25 years	1005.8	33	1.77 (1.19, 2.65)		1005.8	16	2.11 (1.16, 3.83)
Current smokers, < 25 years	416	13	1.41 (0.78, 2.55)		416	8	1.14 (0.57, 2.29)
Current smokers, ≥ 25 years	1617.4	57	1.64 (1.19, 2.26)		1617.4	28	1.50 (0.94, 2.38)
**Intensity of Smoking** [a]							
Never smokers	7796.7	147	Reference		7796.7	85	Reference
Former smokers, < 8 cigarettes/day	1556.7	35	1.21 (0.83, 1.77)		1556.7	18	1.32 (0.77, 2.27)
Former smokers, ≥ 8 cigarettes/day	1671.2	41	1.37 (0.96, 1.96)		1671.2	19	1.38 (0.80, 2.39)
Current smokers, < 8 cigarettes/day	964.4	27	1.31 (0.85, 2.01)		964.4	17	1.40 (0.82, 2.42)
Current smokers, ≥ 8 cigarettes/day	1069.1	43	1.80 (1.26, 2.57)		1069.1	19	1.36 (0.79, 2.32)
**Recency of cessation** [b]							
Never smokers	6787	118	Reference		6787	67	Reference
Current smokers	1792.7	59	1.63 (1.19, 2.25)		1792.7	28	1.33 (0.83, 2.11)
Former smokers, ≤10 years	849.2	21	1.39 (0.87, 2.23)		849.2	10	1.39 (0.67, 2.89)
Former smokers, >10 years	1860.7	41	1.22 (0.85, 1.76)		1860.7	21	1.37 (0.81, 2.33)

HR = Hazard Ratio, 95% CI = 95% Confidence Interval

[a] Cox-proportional hazard models adjusted for age at diagnosis, tumor stage, body mass index (BMI), alcohol consumption, education, household income, marital status, menopausal status, and physical activity. Competing risk models (Fine and Gray subdistribution hazard model) were used for breast cancer-specific mortality.

© 2023 Zeinomar N et al. *JAMA Network Open.*

[b] Cox-proportional hazard models adjusted for age at diagnosis, tumor stage, waist to hip ratio, history of diabetes, history of hypertension, education, household income, and physical activity. Competing risk models (Fine and Gray subdistribution hazard model) were used for breast cancer-specific mortality

© 2023 Zeinomar N et al. *JAMA Network Open.*

eTable 2. Hazard Ratios and 95% Confidence Intervals for Associations of Cigarette Smoking and Mortality After First Breast Cancer in the Women's Circle Of Health Follow-up Study (WCHFS), Excluding Ductal Carcinoma In-Situ

	**All-cause mortality**				**Breast cancer–specific mortality**		
	Person-years	Total deaths	HR (95% CI)		Person-years	Breast cancer deaths	HR (95% CI)
**Smoking Status** [a]							
Never smokers	6371.3	160	Reference		6371.3	101	Reference
Former smokers	2463.5	74	1.24 (0.93, 1.65)		2463.5	40	1.20 (0.81, 1.80)
Current smokers	1789.9	78	1.55 (1.16, 2.07)		1789.9	42	1.28 (0.88, 1.88)
**Pack years of smoking** [a]							
Never smokers	6371.3	160	Reference		6371.3	101	Reference
Former smokers, < 10 pack years	1455.7	45	1.20 (0.85, 1.68)		1455.7	26	1.18 (0.75, 1.86)
Former smokers, ≥ 10 pack years	1007.8	29	1.33 (0.88, 2.01)		1007.8	14	1.26 (0.68, 2.35)
Current smokers, < 10 pack years	775.2	23	1.11 (0.71, 1.75)		775.2	15	1.08 (0.63, 1.84)
Current smokers, ≥ 10 pack years	1014.7	55	1.85 (1.34, 2.56)		1014.7	27	1.43 (0.89, 2.29)
**Duration of smoking** [a]							
Never smokers	6371.3	160	Reference		6371.3	101	Reference
Former smokers, < 25 years	1725.9	43	1.05 (0.75, 1.49)		1725.9	23	0.99 (0.61, 1.61)
Former smokers, ≥ 25 years	737.6	31	1.69 (1.13, 2.55)		737.6	17	1.90 (1.08, 3.35)
Current smokers, < 25 years	401.3	17	1.49 (0.88, 2.52)		401.3	8	0.81 (0.40, 1.68)
Current smokers, ≥ 25 years	1388.6	61	1.58 (1.16, 2.16)		1388.6	34	1.50 (0.98, 2.29)
**Intensity of Smoking** [a]							
Never smokers	6371.3	160	Reference		6371.3	101	Reference
Former smokers, < 8 cigarettes/day	1175.2	37	1.21 (0.84, 1.76)		1175.2	21	1.22 (0.75, 1.98)
Former smokers, ≥ 8 cigarettes/day	1288.3	37	1.26 (0.87, 1.83)		1288.3	19	1.19 (0.69, 2.06)
Current smokers, < 8 cigarettes/day	856.3	30	1.38 (0.91, 2.07)		856.3	19	1.36 (0.83, 2.24)
Current smokers, ≥ 8 cigarettes/day	933.6	48	1.68 (1.20, 2.35)		933.6	23	1.23 (0.75, 2.02)
**Recency of cessation** [b]							
Never smokers	5928	133	Reference		5928	81	Reference
Current smokers	1622.4	63	1.54 (1.13, 2.09)		1622.4	32	1.20 (0.79, 1.84)
Former smokers, ≤10 years	655	20	1.27 (0.79, 2.05)		655	11	1.26 (0.64, 2.51)
Former smokers, >10 years	1545.3	44	1.24 (0.87, 1.77)		1545.3	23	1.22 (0.74, 2.00)

HR = Hazard Ratio, 95% CI = 95% Confidence Interval

[a] Cox-proportional hazard models adjusted for age at diagnosis, tumor stage, body mass index (BMI), alcohol consumption, education, household income, marital status, menopausal status, and physical activity. Competing risk models (Fine and Gray subdistribution hazard model) were used for breast cancer-specific mortality.

© 2023 Zeinomar N et al. *JAMA Network Open.*

[b] Cox-proportional hazard models adjusted for age at diagnosis, tumor stage, waist to hip ratio, history of diabetes, history of hypertension, education, household income, and physical activity. Competing risk models (Fine and Gray subdistribution hazard model) were used for breast cancer-specific mortality

© 2023 Zeinomar N et al. *JAMA Network Open.*
