# Supplementary material for: Association of Cigarette Smoking and Alcohol Consumption With Subsequent Mortality Among Black Breast Cancer Survivors in New Jersey
Source: JAMA Netw Open. 2023 Jan 24;6(1):e2252371. doi: 10.1001/jamanetworkopen.2022.52371 (PMC10148653; doi:10.1001/jamanetworkopen.2022.52371)
Supplement: Supplement 2. — Data Sharing Statement [file jamanetwopen-e2252371-s002.pdf]

Data Sharing Statement

Zeinomar. Association of Cigarette Smoking and Alcohol Consumption With Subsequent Mortality Among Black Breast Cancer Survivors in New Jersey. *JAMA Netw Open*. Published January 24, 2023. doi:10.1001/jamanetworkopen.2022.52371

Data
Data available: Yes
Data types: Other (please specify)
Additional Information: The de-identified data underlying this article can be shared upon approval of a data request form by the Women's Circle of Health Follow-up Study Scientific Committee and with appropriate human subjects approval and data transfer agreements.
How to access data: wchstudy@cinj.rutgers.edu
When available: With publication

Supporting Documents
Document types: None

Additional Information
Who can access the data: Researchers whose proposed use of the data has been approved.
Types of analyses: For approved analyses
Mechanisms of data availability: With a signed data access agreement.
